# The efficacy of acupuncture for trigeminal neuralgia: an overview of systematic reviews

**DOI:** 10.3389/fneur.2024.1375587

**Published:** 2024-07-05

**Authors:** Hong-xian He, Ya-xin Li, Ya-song Xiao, Wen-hui Fan, Hua Xue

**Affiliations:** ^1^Department of Rehabilitation, Sichuan Taikang Hospital, Chengdu, Sichuan, China; ^2^Department of Neurology, Sichuan Taikang Hospital, Chengdu, Sichuan, China; ^3^Department of Geriatrics, Sichuan Taikang Hospital, Chengdu, Sichuan, China

**Keywords:** trigeminal neuralgia, acupuncture, systematic review, grade, AMSTAR-2

## Abstract

**Background:**

Many systematic reviews (SRs) and meta-analysis (MAs) have reported the efficacy of acupuncture treatment for primary trigeminal neuralgia (PTN), but the quality of evidence is unknown and therefore needs to be evaluated comprehensively.

**Methods:**

Eight electronic databases were searched from their inception until January 5, 2024. The methodological quality, reporting quality, and risk of bias of the included SRs were assessed by the assessment of multiple systematic reviews 2 (AMSTAR-2), the Risk of Bias in Systematic Reviews (ROBIS) tool, and the Preferred Reporting Items for Systematic Reviews and Meta-Analyses (PRISMA). The quality of evidence for outcome measures was evaluated using the Grading of Recommendations Assessment, Development, and Evaluation (GRADE).

**Results:**

We identified 13 SRs/MAs met inclusion criteria. According to the results of the AMSTAR-2, six were rated as critically low quality and seven as low quality. According to ROBIS assessment, 8 SRs/MAs were classified as low risk, and 5 SRs/MAs were found to be high risk. The PRISMA report still has some reporting deficiencies in aspects such as protocol and registration, search strategy, risk of bias, additional analyzes and funding. According to the GRADE system, no high-quality evidence was found, 1 was of moderate quality, 4 were of low quality, and 8 were of critical low quality.

**Conclusion:**

Based on the evidence collected, acupuncture shows promise as a treatment for PTN patients. However, it is important to note that the included SRs/MAs generally have low methodological quality and evidence quality. Therefore, caution must be exercised when interpreting this conclusion. To enhance future research in this area, it is recommended to adequately report methodological details and adhere to guidelines for conducting SRs/MAs.

**Systematic review registration**: https://www.crd.york.ac.uk/prospero/, identifier CRD42024499280.

## 1 Introduction

Primary trigeminal neuralgia (PTN) is a prevalent neurological disease characterized by recurrent, brief episodes of intense pain in the trigeminal nerve distribution area (1). The pain is often described as electric shock-like, burning, tearing, or knife-like. It typically affects one side of the face, but can also occur bilaterally (2). Epidemiological studies have shown that PTN is 1.5 per 10,000 people and an annual incidence rate of 0.3–0.5 per 10,000 people (3). The onset of PTN can range from 28 to 89 years old, with 70 to 80% of cases occurring in individuals over 40 years old. The highest prevalence is observed between the ages of 50 and 60 (4). Over time, the frequency of trigeminal nerve pain increases, significantly impacting the mental health of patients. Patients often experience accompanying symptoms such as anxiety, depression, and sleep disorders (5).

The etiology and pathogenesis of PTN remain unclear. Currently, two prominent theories are recognized: the trigeminal microvascular compression theory and the trigeminal nerve demyelination theory (6). Treatment options for PTN mainly include drug therapy, nerve block, radiofrequency thermocoagulation, and surgical treatment (7). Carbamazepine and oxcarbazepine are commonly used drugs for the treatment of PTN, which is based on the principle of reducing or blocking the transmission of pain signals by inhibiting abnormal neuronal discharge (7–10). Carbamazepine is more effective in the treatment of PTN and can relieve pain symptoms. However, long-term use may lead to the development of drug resistance and some side effects such as dizziness, drowsiness, nausea, vomiting, and more serious ones such as drug-induced hepatitis, and bone marrow suppression (8). Oxcarbazepine, a ketone-based derivative of carbamazepine, also has antiepileptic and analgesic effects and is indicated for the treatment of a wide range of neuropathic pain including PTN. Compared with carbamazepine, oxcarbazepine has a lower incidence of side effects, such as drowsiness and vertigo, which are about 1/3 of those of carbamazepine, and serious adverse events are rarer (9). Acupuncture, as a traditional Chinese medicine therapy, has a long history of treating PTN and has good efficacy and safety (11). Systematic reviews (SRs)/Meta-analyses (MAs) are important tools to guide evidence-based clinical practice and have been widely used in various medical fields in recent years. As the highest level of evidence-based evidence, MAs/SRs can provide a basis for clinical decision-making, but low-quality MAs/SRs among them can also mislead clinical decisions (12). Systematic review re-evaluation can comprehensively evaluate the quality of MAs/SRs to guide clinical decision-making (13). This study aimed to use the Methodological Quality Assessment Tool for Systematic Reviews 2 (AMSTAR-2), the Risk of Bias Assessment Tool for Systematic Reviews (ROBIS) and the Prioritized Reporting Items for Systematic Reviews and Meta-Analyses (PRISMA) to evaluate the methodological quality and reporting of included systematic reviews. GRADE (Grading of Recommendations, Assessment, Development, and Evaluation) was used to evaluate the quality of evidence for outcome indicators. It is believed that this study will provide more reliable reference and evidence-based support for the clinical practice and related research of acupuncture treatment of PTN.

## 2 Methods

### 2.1 Registration

A predetermined written protocol of this overview was registered in the International prospective register of systematic overview (PROSPERO) database, registration number: CRD42017077218. We conducted this study based on a high-quality methodological review and the Cochrane Handbook (14).

### 2.2 Inclusion criteria and exclusion criteria

The inclusion criteria were as follows: (a) study design and participants: the SRs/MAs of all randomized controlled trials (RCTs) or quasi-randomized controlled trials (q-RCTs) of acupuncture in PTN will be included in this overview. There are no restrictions on patients’ age, gender, race, or course of disease; (b) intervention: there was no restriction on the types of acupuncture (e.g., body acupuncture, electroacupuncture, ear acupuncture, warm-acupuncture, and scalp acupuncture); (c) comparison: the control group can be conventional treatment, waiting list patients, sham/placebo acupuncture, medication, cognitive rehabilitation training, or other treatments; (d) outcomes: the primary outcome are efficacy rate and visual analog scale (VAS), while the secondary outcomes are frequency of pain attacks, recurrence rate, and adverse events. Network meta-analyses, comments, reviews and meeting abstracts will be excluded. Non-invasive treatment is not considered, such as transcutaneous electrical acupoint stimulation, ear acupoint pressure, moxibustion, acupoint embedding and acupoint injection.

### 2.3 Search strategy

We conducted comprehensive searches from eight databases, including SinoMed, China National Knowledge Infrastructure (CNKI), Wanfang, VIP, Cochrane Library, Embase, PubMed, and Web of Science, respectively, from the inception to January 5, 2024, with no language restrictions. Relevant net terms and text terms were adjusted according to the specific requirements of different databases. In addition, a manual search of relevant references was conducted to identify additional eligible studies. The detailed search strategy is shown in Supplementary Table S1.

### 2.4 Study selection and data extraction

Based on the search strategy, two reviewers independently conducted literature search and screening, and all retrieved studies were imported into EndNote. After removing duplicate articles, two reviewers conducted independent selection based on title and abstract. Then, two reviewers read the full text for further evaluation. Any disputes were resolved by third reviewer. After identifying the eligible studies, two reviewers independently extracted relevant data according to the standardized extraction form, such as author, year of publication, number of RCTs, sample size, intervention measures, outcome indicators, risk assessment tools, adverse reactions, conclusions.

### 2.5 Evaluation methods

Two independent researchers assessed the methodological quality, reporting quality, risk of bias, and evidence quality by the Assessing the Methodological Quality of Systematic Reviews 2 (AMSTAR-2), Preferred Reporting Items for Systematic Reviews and Meta-Analyses (PRISMA), Risk of Bias in Systematic Reviews (ROBIS), and Grading of Recommendations, Assessment, Development, and Evaluation (GRADE), respectively.

The methodological quality of the included studies was evaluated using the AMSTAR-2 scale, which consisted of 16 items (15). The scale assessed various aspects such as study design plan, literature screening, data extraction, search strategy, basic information about the included studies, publication bias, conflict of interest, and funding. Items were categorized as “Yes” if adequately performed, “Partially yes” if correct responses were given based on limited information, and “No” if the project was not evaluated for relevance or was incorrectly evaluated. A quality rating was then assigned based on the grading criteria of the AMSTAR-2 checklist.

Two independent reviewers assessed the reporting quality of the included SRs/MAs by the updated version of the PRISMA checklist (16). The PRISMA checklist consists of 27 items that are considered essential for transparent reporting. Items that were answered correctly and appropriately were classified as “Yes,” indicating accurate and appropriate recording. If the items were answered correctly but based on limited evidence, they were classified as “Partially yes.” However, if the item was not properly assessed or the assessment itself was incorrect, it was classified as “No.”

We used the ROBIS tool to assess the risk of bias (RoB) of SRs/MAs (17). The evaluation process is divided into three stages: evaluating the correlation, determining the degree of RoB in the SRs/MAs process, and judging RoB. The four domains of the second stage including study eligibility criteria, identification and selection of studies, collection and study appraisal, synthesis and funding. The risk of bias can be rated as “low risk,” “high risk,” or “unclear.”

The quality of evidence in the included SR/MAs was assessed using the GRADE method, which is specifically designed to assess the quality of evidence in SR/MAs (18). The GRADE system categorizes the quality of evidence into four grades, namely, “high,” “moderate,” “low” and “critical low.” The initial rating is reduced if there are study limitations, inconsistencies, imprecision, indirectness or publication bias.

## 3 Results

### 3.1 Results on literature search and selection

**Figure 1:**
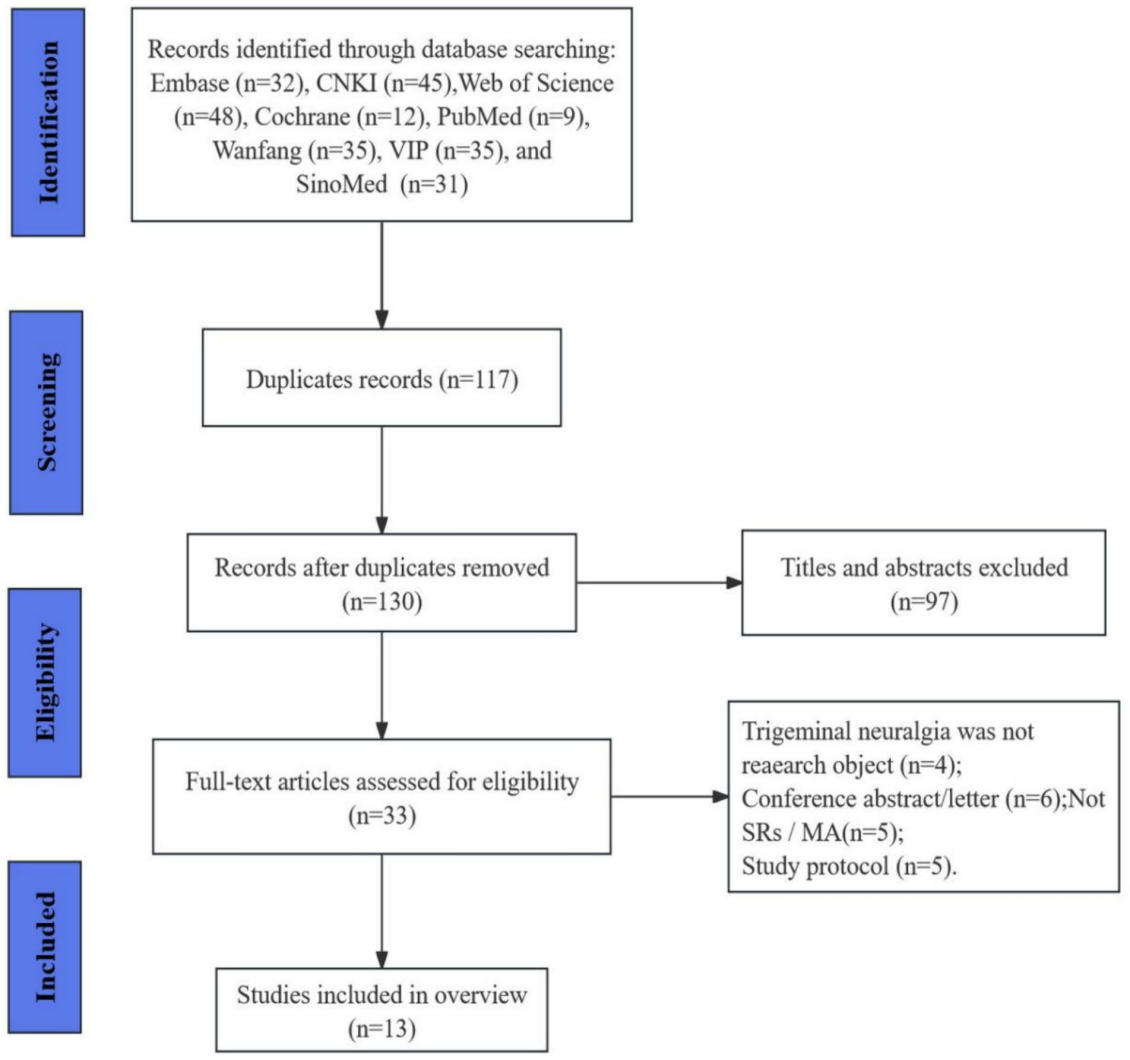
The flowchart of the literature selection.

We conducted a search and retrieved 247 publications based on the search strategy from the eight databases. After screening the titles and abstracts, 117 duplicates and 97 ineligible studies were excluded. The remaining 33 articles were identified to be of interest. After full-text review, 20 articles were excluded due to not being SRs/MAs (*n* = 5), PTN was not research object (*n* = 4), conference/letter (*n* = 6), and protocol (*n* = 5). Thus, 13 reviews met the inclusion criteria and were included in the final analysis. The complete screening and selection process is presented visually in Figure 1.

### 3.2 Characteristics of included SRs

The characteristics of the 13 SRs/MAs included in our final analysis are summarized in Table 1 (19–31). They were published between 2009 and 2023, 10 of them were written in Chinese, and the remaining three were written in English. The SRs comprised a total of 223 RCTs and 15,911 subjects. The number of RCTs included in the SRs/MAs ranged from 8 to 35, with sample sizes ranging from 477 to 2,295 participants. The intervention in the treatment group was mainly acupuncture or acupuncture combined with conventional treatment, while in the control group, conventional treatment or sham acupuncture was used. In terms of quality assessment scales, two used Jadad and the others used Cochrane risk of bias criteria. Among the 13 SR/MAs, 3 were published in English, and all SR/MAs reached positive conclusions.

**Table 1:** Characteristics of the included reviews.

Included studies	No. of RCTs	Participants	Experimental intervention	Control intervention	Risk assessment tools	Meta-analyses	Adverse effects (no. of cases)	Conclusion summary	Results summary
Ang et al. (19)	30	2,295	Acupuncture	Conventional therapy	Cochrane risk of bias tool	Yes	E: 3 C: 7	Acupuncture may improve trigeminal neuralgia-related pain	Positive
Fang et al. (20)	15	1,255	Acupuncture	Conventional therapy	Cochrane risk of bias tool	Yes	Not reported	Acupuncture and moxibustion treatment for primary trigeminal neuralgia was safe and effective	Positive
Gao et al. (21)	8	885	Acupuncture	Conventional therapy	Cochrane risk of bias tool	Yes	Not reported	Acupuncture treatment of trigeminal neuralgia is safe and effective	Positive
Gao and Wei (22)	12	958	Acupuncture	Conventional therapy	Jadad	Yes	23	Acupuncture treatment of trigeminal neuralgia curative effect is better than western medicine, and less adverse reactions.	Positive
Hu et al. (23)	33	1,389	Acupuncture	Conventional therapy	Cochrane risk of bias tool	Yes	Not reported	Acupuncture might have some positive effects for primary trigeminal neuralgia	Positive
Liu et al. (24)	12	920	Acupuncture	Conventional therapy	Cochrane risk of bias tool	No	Not reported	Acupuncture treatment of trigeminal neuralgia is safe and effective	Positive
Liu (25)	35	2,512	Acupuncture	Conventional therapy	Cochrane risk of bias tool	Yes	E: 4 C: 37	Among the acupuncture related therapies of primary trigeminal neuralgia, acupuncture therapy was the best, and acupuncture combined with carbamazepine, all of which were better than the treatment of carbamazepine alone.	Positive
Ren (26)	16	1,172	Acupuncture	Conventional therapy	Cochrane risk of bias tool	Yes	E: 2 C: 6	There is clinical evidence that acupuncture treatment of primary trigeminal neuralgia is a safe and effective method is superior to the existing effective drugs.	Positive
Wang et al. (27)	11	891	Acupuncture	Conventional therapy	Cochrane risk of bias tool	Yes	Not reported	Acupuncture was a safe and effective therapy for primary trigeminal neuralgia	Positive
Wu et al. (28)	8	477	Acupuncture	Conventional therapy	Cochrane risk of bias tool	Yes	Not reported	Electroacupuncture is effective for primary trigeminal neuralgia	Positive
Yin et al. (29)	16	1,231	Acupuncture	Conventional therapy	Cochrane risk of bias tool	Yes	E: 8 C: 36	Acupuncture treatment of trigeminal neuralgia is superior to carbamazepine in terms of analgesia, and is safer than carbamazepine.	Positive
Zhang (30)	15	1,119	Acupuncture	Conventional therapy	Jadad	Yes	Not reported	Acupuncture treatment of trigeminal neuralgia is effective	Positive
Zhou et al. (31)	12	807	Acupuncture	Conventional therapy	Cochrane risk of bias tool	Yes	Not reported	The total efficacy rate for trigeminal neuralgia is higher in the electroacupuncture group than in the control group	Positive

### 3.3 Results of the methodological quality

According to AMSTER-2, Table 2 provides an overview of the methodological quality assessed in the systematic evaluation. Out of the 13 SRs, 6 were rated as very low quality and 7 as low quality. All SRs followed the principles of the PICO (P: study population; I: intervention; C: control; O: outcome) research questions and inclusion criteria. They also employed a comprehensive literature search strategy and provided essential characteristics of each RCT. Four SRs were pre-registered for the study protocol (19, 23, 25, 29). Additionally, all SRs explicitly stated that literature screening and data extraction were conducted independently by two individuals. Appropriate tools, such as the Cochrane Risk of Bias Tool and the Jadad Scale, were used in assessing the possible risk of bias in the included RCTs. Six SRs reported their funding sources. However, not all meta-analyses considered the potential impact of risk of bias on the overall effect in RCTs. Overall, the methodological quality of the included SRs was deemed unsatisfactory.

**Table 2:** Methodological quality of included SRs on acupuncture for primary trigeminal neuralgia.

Included studies	AMSTAR 2																Overall quality
	Q 1	Q 2	Q 3	Q 4	Q 5	Q 6	Q 7	Q 8	Q 9	Q 10	Q 11	Q 12	Q 13	Q 14	Q 15	Q 16	
Ang et al. (19)	Y	Y	Y	Y	Y	Y	Y	Y	Y	Y	Y	Y	Y	Y	Y	Y	CL
Fang et al. (20)	Y	N	Y	PY	Y	Y	N	PY	PY	Y	Y	N	N	N	N	Y	L
Gao et al. (21)	Y	N	Y	PY	Y	Y	N	PY	PY	Y	Y	Y	Y	N	N	Y	L
Gao and Wei (22)	Y	N	Y	PY	Y	Y	Y	Y	Y	N	Y	Y	Y	N	N	Y	L
Hu et al. (23)	Y	Y	Y	Y	Y	Y	Y	Y	Y	N	Y	Y	Y	N	N	Y	CL
Liu et al. (24)	Y	N	Y	PY	Y	Y	Y	PY	PY	N	Y	Y	Y	N	N	Y	L
Liu (25)	Y	Y	Y	Y	Y	Y	Y	Y	Y	N	Y	Y	Y	Y	N	Y	CL
Ren (26)	Y	N	Y	PY	Y	Y	Y	PY	Y	N	Y	Y	Y	N	N	Y	L
Wang et al. (27)	Y	N	Y	PY	Y	Y	N	PY	Y	N	N	N	N	N	N	Y	L
Wu et al. (28)	Y	N	Y	Y	Y	Y	Y	Y	Y	Y	Y	Y	Y	Y	N	Y	CL
Yin et al. (29)	Y	Y	Y	Y	Y	Y	Y	Y	Y	Y	Y	Y	Y	Y	N	Y	CL
Zhang (30)	Y	N	Y	PY	Y	Y	N	Y	PY	N	Y	Y	Y	N	N	Y	L
Zhou et al. (31)	Y	N	Y	Y	Y	Y	Y	Y	Y	Y	Y	Y	N	PY	N	Y	CL

The key items of the AMSTAR 2. H: represents the ranking of quality as high; M: represents the ranking of quality as moderate; L: represents the ranking of quality as low; CL: represents the ranking of quality as critically low. Q1: did the research questions and inclusion criteria for the review include the components of PICO? Q2: did the report of the review contain an explicit statement that the review methods were established prior to the conduct of the review and did the report justify any significant deviations from the protocol? Q3: did the review authors explain their selection of the study designs for inclusion in the review? Q4: did the review authors use a comprehensive literature search strategy? Q5: did the review authors perform study selection in duplicate? Q6: did the review authors perform data extraction in duplicate? Q7: did the review authors provide a list of excluded studies and justify the exclusions? Q8: did the review authors describe the included studies in adequate detail? Q9: did the review authors use a satisfactory technique for assessing the risk of bias (RoB) in individual studies that were included in the review? Q10: did the review authors report on the sources of funding for the studies included in the review? Q11: if a meta-analysis was performed, did the review authors use appropriate methods for the statistical combination of results? Q12: if a meta-analysis was performed, did the review authors assess the potential impact of RoB in individual studies on the results of the meta-analysis or another evidence synthesis? Q13: did the review authors account for RoB in primary studies when interpreting/discussing the results of the review? Q14: did the review authors provide a satisfactory explanation for, and discussion of, any heterogeneity observed in the results of the review? Q15: if they performed quantitative synthesis did the review authors carry out an adequate investigation of publication bias (small study bias) and discuss its likely impact on the results of the review? Q16: did the review authors report any potential sources of conflict of interest, including any funding they received for conducting the review? AMSTAR 2, Assessment of Multiple Systematic Reviews 2; N, no; PY, partial yes; Y, yes.

### 3.4 Results of the reporting quality

Table 3 presents the quality components of the PRISMA checklist for acupuncture treatment PTN. The overall report is not comprehensive, and all SRs reports discuss 10 items. These items include title, structured summary, objectives, eligibility criteria, information sources, search, summary measures, study selection, limitations, and conclusions. However, there are some shortcomings. In the “Methods” section, only 4 out of 13 SRs (30.76%) reported the protocol and registration status (19, 23, 25, 29). In the “Results” section, 12 SRs provided a thorough explanation of the study characteristics, risk of bias within studies, and synthesis of results (92.31%) (19–23, 25–31). Eleven SRs reported the results of individual studies and risk of bias across studies, while only 7 SRs reported additional analysis (53.84%). In the “Discussion” section, all SRs addressed limitations and conclusions. Regarding funding, only 61.53% of studies reported funding (19–21, 23, 28–31). Overall, the reporting quality of the included SRs was relatively low.

**Table 3:** Results of the PRISMA for the acupuncture checklist.

Section/topic	Items	Ang et al. (19)	Fang et al. (20)	Gao et al. (21)	Gao and Wei (22)	Hu et al. (23)	Liu et al. (24)	Liu (25)	Ren (26)	Wang et al. (27)	Wu et al. (28)	Yin et al. (29)	Zhang (30)	Zhou et al. (31)	Compliance (%)
Title	Q1. Title	Y	Y	Y	Y	Y	Y	Y	Y	Y	Y	Y	Y	Y	100
Abstract	Q2. Structured summary	Y	Y	Y	Y	Y	Y	Y	Y	Y	Y	Y	Y	Y	100
Introduction	Q3. Rationale	Y	N	Y	N	Y	Y	Y	Y	N	Y	Y	N	N	61.53
	Q4. Objectives	Y	Y	Y	Y	Y	Y	Y	Y	Y	Y	Y	Y	Y	100
Methods	Q5. Protocol and registration	Y	N	N	N	Y	N	Y	N	N	N	Y	N	N	30.76
	Q6. Eligibility criteria	Y	Y	Y	Y	Y	Y	Y	Y	Y	Y	Y	Y	Y	100
	Q7. Information sources	Y	Y	Y	Y	Y	Y	Y	Y	Y	Y	Y	Y	Y	100
	Q8. Search	Y	Y	Y	Y	Y	Y	Y	Y	Y	Y	Y	Y	Y	100
	Q9. Study selection	Y	Y	Y	Y	Y	N	Y	Y	Y	Y	Y	Y	Y	92.31
	Q10. Data collection process	Y	Y	Y	Y	Y	N	Y	Y	Y	Y	Y	Y	Y	92.31
	Q11. Data items	Y	Y	Y	Y	Y	N	Y	Y	Y	Y	Y	Y	Y	92.31
	Q12. Risk of bias in individual studies	Y	Y	Y	Y	Y	N	Y	Y	Y	Y	Y	Y	Y	92.31
	Q13. Summary measures	Y	Y	Y	Y	Y	Y	Y	Y	Y	Y	Y	Y	Y	100
	Q14. Synthesis of results	Y	N	N	Y	Y	N	Y	Y	Y	Y	Y	Y	Y	76.92
	Q15. Risk of bias across studies	Y	Y	Y	Y	Y	N	Y	Y	Y	Y	Y	Y	Y	92.31
	Q16. Additional analyses	Y	N	Y	Y	Y	N	Y	Y	N	Y	Y	Y	Y	76.92
Results	Q17. Study selection	Y	Y	Y	Y	Y	Y	Y	Y	Y	Y	Y	Y	Y	100
	Q18. Study characteristics	Y	Y	Y	Y	Y	N	Y	Y	Y	Y	Y	Y	Y	92.31
	Q19. Risk of bias within studies	Y	Y	Y	Y	Y	N	Y	Y	Y	Y	Y	Y	Y	92.31
	Q20. Results of individual studies	Y	Y	Y	Y	Y	N	Y	Y	N	Y	Y	Y	Y	84.61
	Q21. Synthesis of results	Y	Y	Y	Y	Y	N	Y	Y	Y	Y	Y	Y	Y	92.31
	Q22. Risk of bias across studies	Y	Y	Y	Y	Y	N	Y	Y	N	Y	Y	Y	Y	84.61
	Q23. Additional analysis	Y	N	N	N	Y	N	Y	Y	N	Y	Y	N	Y	53.84
Discussion	Q24. Summary of evidence	Y	Y	Y	Y	Y	N	Y	Y	Y	Y	Y	Y	Y	92.31
	Q25. Limitations	Y	Y	Y	Y	Y	Y	Y	Y	Y	Y	Y	Y	Y	100
	Q26. Conclusions	Y	Y	Y	Y	Y	Y	Y	Y	Y	Y	Y	Y	Y	100
Funding	Q27. Funding	Y	Y	Y	N	Y	N	N	N	N	Y	Y	Y	Y	61.53

N, no; PY, partial yes; Y, yes.

### 3.5 Risk of bias of included SRs

Table 4 displays the results of the risk of bias assessment of the included studies using the ROBIS tool. In Stage 1, the relevance of the study topic was evaluated, and all SRs/MAs were rated as having a low risk of bias, indicating their relevance to the study topic. Stage 2 consisted of four domains. All included SRs/MAs were assessed as having a low risk in the study eligibility criteria domains. In the identification and selection of studies, eight SR/MAs were assessed as low risk, while five SRs/MAs were rated as high risk due to a lack of searches in clinical trial registries and other databases. Additionally, four SRs/MAs were classified as high risk due to a lack of detailed description of study bias evaluation in the collection and study assessment (20, 21, 24, 30). The synthesis and findings domains revealed that five SRs/MAs were categorized as high risk because sensitivity analyses were not performed to assess the stability of the results, while the remaining SR/MAs were considered low risk. In Stage 3, considering the overall risk of bias, five SRs/MAs were categorized as high risk for not providing explanations or additions regarding the risk of bias, while the remaining SR/MAs were considered low risk.

**Table 4:** Risk of bias of the included SRs.

Included Studies	Phase 1	Phase 2				Phase 3
	Assessing relevance	Domain 1: study eligibility criteria	Domain 2: identification and selection of studies	Domain 3: collection and study appraisal	Domain 4: synthesis and findings	Risk of bias in the review
Ang et al. (19)	Low risk	Low risk	Low risk	Low risk	Low risk	Low risk
Fang et al. (20)	Low risk	Low risk	High risk	High risk	High risk	High risk
Gao et al. (21)	Low risk	Low risk	High risk	High risk	High risk	High risk
Gao and Wei (22)	Low risk	Low risk	High risk	Low risk	High risk	High risk
Hu et al. (23)	Low risk	Low risk	Low risk	Low risk	Low risk	Low risk
Liu et al. (24)	Low risk	Low risk	High risk	High risk	High risk	High risk
Liu (25)	Low risk	Low risk	Low risk	Low risk	Low risk	Low risk
Ren (26)	Low risk	Low risk	Low risk	Low risk	Low risk	Low risk
Wang et al. (27)	Low risk	Low risk	Low risk	Low risk	Low risk	Low risk
Wu et al. (28)	Low risk	Low risk	Low risk	Low risk	Low risk	Low risk
Yin et al. (29)	Low risk	Low risk	Low risk	Low risk	Low risk	Low risk
Zhang (30)	Low risk	Low risk	High risk	High risk	High risk	High risk
Zhou et al. (31)	Low risk	Low risk	Low risk	Low risk	Low risk	Low risk

### 3.6 Evidence quality

The GRADE system evaluated 21 outcomes from 10 MAs. The assessment revealed that there was no high-quality evidence available. Only one outcome presented moderate-quality evidence, while four outcomes had low-quality evidence, and 16 outcomes had very low-quality evidence. The evidence was degraded due to limitations, inconsistency, imprecision, and publication bias in RCTs. For more detailed information, please refer to Table 5.

**Table 5:** Results of evidence quality.

Included studies	Outcomes	Limitations	Inconsistency	Indirectness	Imprecision	Publication bias	Relative effect (95% CI)	*p*-value	Quality
Ang et al. (19)	Pain (visual analog scale)	−1	0	0	−1	0	MD −1.40 (−1.82, −0.98)	<0.05	L
	Effective rate	−1	0	−1	0	0	OR 1.20 (1.15, 1.25)	<0.05	L
	Frequency of pain attacks	−1	0	−1	−1	−1	MD −2.53 (−4.11, −0.96)	<0.05	CL
Fang et al. (20)	Effective rate	−1	0	−1	−1	−1	OR 1.19 (1.13, 1.25)	<0.05	CL
Gao et al. (21)	Effective rate	−1	0	0	−1	−1	OR 4.32 (2.99, 6.22)	<0.01	CL
	Pain (visual analog scale)	−1	0	−1	−1	−1	MD −0.34 (−0.62, −0.06)	<0.02	CL
Gao and Wei (22)	Effective rate	−1	0	0	−1	−1	OR 4.15 (2.76, 6.23)	<0.05	CL
Hu et al. (23)	Effective rate	−1	0	0	−1	0	OR 3.80 (2.82, 5.12)	<0.01	L
	Pain intensity	−1	−1	−1	−1	0	MD −0.58 (−1.91, 0.76)	0.008	CL
	Recurrence rate	−1	−1	0	0	−1	OR 0.43 (0.19, 0.98)	0.04	CL
Liu (25)	Effective rate	−1	0	0	0	0	OR 1.12 (1.07, 1.17)	<0.05	M
	Pain (visual analog scale)	−1	0	−1	−1	−1	MD −0.98 (−1.64, −0.32)	0.003	CL
Ren (26)	Effective rate	−1	0	0	−1	−1	OR 9.68 (6.48, 14.47)	<0.001	CL
Wang et al. (27)	Effective rate	−1	0	0	−1	−1	OR 2.97 (2.07, 4.25)	<0.001	CL
Wu et al. (28)	Effective rate	−1	0	0	−1	0	OR 4.24 (2.35, 7.66)	<0.001	L
	Recurrence rate	−1	0	−1	−1	−1	OR 0.36 (0.11, 1.17)	0.09	CL
Yin et al. (29)	Degree of pain reduction	−1	0	−1	−1	0	MD 1.47 (0.99, 1.95)	<0.001	CL
Zhang (30)	Effective rate	−1	0	0	−1	−1	OR 0.18 (0. 10, 0. 26)	<0.001	CL
Zhou et al. (31)	Effective rate	−1	0	0	0	−1	OR 4.04 (2.67, 6.13)	<0.001	CL
	Pain (visual analog scale)	−1	−1	0	−1	0	MD −0.06 (−0.59, 0.47)	0.82	CL
	Recurrence rate	−1	−1	−1	0	−1	OR 0.64 (0.05, 7.45)	0.72	CL

1, downgrade; 0, not downgrade; CL, critically low; L, low; M, moderate; H, high.

### 3.7 Outcomes

#### 3.7.1 Effective rate

A total of 11 SRs analyzed the effectiveness of acupuncture in treating PTN, and all studies demonstrated that acupuncture was more effective in treating PTN than the control groups. Specifically, 11 articles documented the overall efficacy rate: odds ratio (OR) = 1.20, 95% confidence interval (CI): 1.15 ~ 1.25, *p* < 0.05; OR = 1.19, 95% CI: 1.13 ~ 1.25, *p* < 0.05; OR = 4.32, 95% CI: 2.99 ~ 6.22, *p* < 0.01; OR = 4.15, 95% CI: 2.76 ~ 6.23, *p* < 0.05; OR = 3.80, 95% CI: 2.82 ~ 5.12, *p* < 0.01; OR = 1.12, 95% CI: 1.07 ~ 1.17, *p* < 0.05; OR = 9.68, 95% CI: 6.48 ~ 14.47, *p* < 0.001; OR = 2.97, 95% CI: 2.07 ~ 4.25, *p* < 0.001; OR = 4.24, 95% CI: 2.35 ~ 7.66, *p* < 0.001; OR = 0.18, 95% CI: 0. 10 ~ 0. 26, *p* < 0.001; OR = 4.04, 95% CI: 2.67 ~ 6.13, *p* < 0.001.

#### 3.7.2 Pain (visual analog scale)

Four SRs assessed VAS to compare the effect of acupuncture in the treatment of PTN, and three SRs observed that acupuncture has a significant effect on PTN (MD = −1.40, 95% CI [−1.82 ~ −0.98], *p* < 0.05; MD = −0.34, 95% CI [−0.62 ~ −0.06], *p* < 0.02; MD = −0.98, 95% CI [−1.64 ~ −0.32], *p* = 0.003) (19, 21, 25). However, one SRs found that the difference in VAS scores of acupuncture treatment for PTN was not statistically significant (MD = −0.06, 95% CI [−0.59 ~ 0.47], *p* = 0.82) (31).

#### 3.7.3 Recurrence rate

Three studies reported the recurrence rate of acupuncture in the treatment of PTN, and two SRs studies showed that there was no significant difference in the recurrence rate between the acupuncture group and the western medicine group (MD = 0.43, 95% CI [0.19 ~ 0.98], *p* = 0.04; MD = 0.36, 95% CI [0.11 ~ 1.17], *p* = 0.09; MD = 0.64, 95% CI [0.05 ~ 7.45], *p* = 0.72) (23, 28, 31).

#### 3.7.4 Adverse events

Five SRs reported adverse events, and eight SRs did not mention adverse events (19, 22, 25, 26, 29). Adverse reactions in the intervention group was significantly less than the control group. The intervention group was mainly manifested as pigmentation, fainting and drowsiness. The control group mainly showed dizziness, exfoliative dermatitis, rash, vomiting, stomach distension and other discomfort.

## 4 Discussion

The SRs/MAs is a rigorous and comprehensive approach that collects and evaluates existing clinical studies on a specific clinical question, such as the causes, diagnosis, treatment, and prognosis of a disease (17). By applying strict evaluation criteria, the literature is carefully screened to ensure quality, and then qualitatively or quantitatively synthesized to draw reliable conclusions. High-quality SRs/MAs is crucial for ensuring the validity, clarity, and accurate interpretation of evidence (32). In recent years, there has been an increase in the number of SRs/MAs focusing on acupuncture for PTN. However, the quality of these studies varies, and there are limitations in their findings. Conducting comprehensive overview of SRs/MAs that reevaluate existing SRs/MAs related to the same disease or health problem allows for a more thorough integration of evidence, resulting in higher-quality evidence for clinicians. To the best of our knowledge, this review is the first study to comprehensively evaluate SRs/MAs of acupuncture for PTN and has identified some key findings.

We conducted a comprehensive analysis of 13 SRs/MAs in acupuncture treated PTN patients, involving 223 RCTs and 15,911 participants. Our overview revealed unsatisfactory methodological quality, reporting quality, risk of bias, and quality of evidence in the included SRs/MAs. Out of the 13 SRs/MAs, 6 were considered to be of very low quality and 7 were considered to be of low quality according to AMSTAR-2 criteria. These SRs/MAs particularly lacked proper protocol registration, literature search strategy, funding sources, additional analyses, and consideration of publication bias. Insufficient reporting was observed in areas such as protocol registration, risk of bias assessment, additional analyses, and risk of publication bias. In terms of risk of bias assessment using ROBIS, almost all included SRs/MAs were rated as high risk at stage 2, indicating an increased risk of bias. GRADE assessment revealed no high-quality evidence, with risk of bias being the most common factor leading to downgrading of evidence, followed by imprecision, inconsistency, publication bias, and indirectness. The assessment results from these tools highlight common areas that require improvement in the included SRs/MAs. Moving on to the analysis of clinical efficacy, we compared the clinical effective rate, visual analog scale (VAS) score, and recurrence rate of acupuncture treatment for PTN. The results demonstrated that the intervention group had significantly better effective rate and VAS score compared to the control group. However, it is important to note that the methodological quality of all SRs/MAs was rated as low or critical low, and the GRADE results indicated a need for improvement in the quality of evidence for the efficacy of acupuncture.

The pathogenesis of PTN is currently not well understood in the field of modern medicine. It is believed to primarily be a sensory epileptic seizure caused by local mechanical compression of the trigeminal nerve, demyelination, and ectopic impulses resulting from various factors (33, 34). This can create a short circuit in touch forms, transmitting slight pain stimulation to the center and causing severe pain. Three main hypotheses have been proposed. Devor et al. (35) proposed the “ignition” hypothesis, suggesting that damage to the trigeminal nerve root triggers stimulus-induced bursts of electrical activity in some of the damaged neurons, causing them to become overexcited and susceptible to crossover excitation due to the close proximity of neurons to the site of nerve root compression, which ultimately leads to trigeminal neuralgia. Jannetta et al. (36) proposed the doctrine of neurovascular compression, which suggests the presence of compression at the entry of the trigeminal nerve root into the brainstem and at the exit of the trigeminal nerve, including simple contact with the nerve or the presence of a marked depression, nerve atrophy, nerve twisting, and malformation. The third hypothesis is the central neuropathy hypothesis, which states that the sensory disturbances (e.g., persistent pain, dysesthesia, or hypesthesia) seen in patients with trigeminal neuralgia may be related to a brainstem lesion that most likely involves the caudal subnucleus of the nucleus of the spinal tract of the trigeminal nerve, and that there is a correlation between these structures and the duration of trigeminal neuralgia (37). Acupuncture is commonly used in China to treat various conditions, such as infertility, chronic pain, insomnia, and mental illness (38, 39). It has been found that acupuncture can enhance the release of endogenous analgesic substances in the body, raise the pain threshold, and improve local circulation to aid in the dissipation of pain-causing metabolites, ultimately providing analgesic effects (40). The mechanism behind acupuncture analgesia is largely attributed to neuro-humoral factors, which serve as the material basis for the analgesic effects of acupuncture (41). Acupuncture information counteracts the perception and transmission of nociceptive information at all levels, from the periphery to the central nervous system, including acupoint receptors, peripheral afferent pathways, the central nervous system, and central neurotransmitters. This activation also stimulates opioid peptides, 5-HT, norepinephrine, and other biologically active substances to inhibit the sensitivity of peripheral nociceptors and reduce peripheral and central pro-inflammatory cytokines (42, 43). Research has demonstrated that applying 2 Hz electroacupuncture at the Zusanli point can raise the mechanical pain threshold and thermal pain threshold in rats with neuropathic pain (44). Additionally, it has been found to significantly suppress nerve injury-induced metallopeptidase-9, metallopeptidase-2, TNF-α, and highly expressed IL-6 and IL-1β in the spinal cord. Electroacupuncture achieves a reduction in neuropathic pain by activating 5-HT1A receptors and inhibiting *N*-methyl-d-aspartate (NMDA) receptor activity (45). These chemicals, when elevated in the spinal cord, facilitate the transmission of noxious information. Therefore, the analgesic effect of acupuncture may be attributed to the inhibition of their expression.

Currently, the effectiveness of acupuncture in treating PTN is receiving increasing attention, and SR based on high-quality RCTs is crucial for clinical decision-making in evidence-based medicine. However, the significant increase in the number of SRs/MAs has raised questions about their quality. To our knowledge, this is the first time that different research reports have been comprehensively assessed through a comprehensive search and using internationally recognized assessment tools. We conducted a systematic assessment of the methodological quality, risk of bias, and evidence quality of relevant SRs using AMSTAR2, PRISMA, ROBIS, and GRADE tools, respectively. We can intuitively understand the overall quality of SR and the reliability of the results. It is important to recognize that our review has some limitations: (1) We could only synthesize and quantitatively describe the available data. (2) Differences in RCT study design and details of acupuncture interventions may lead to a high RoB for SRs, which in turn affects the quality of evidence and methodological rigor. In trials of acupuncture treatment, it is difficult to achieve double-blindness (i.e., neither patients nor investigators are aware of the subgroups), which may lead to assessment bias. (3) Insufficient sample size may result in trials failing to detect differences that actually exist (i.e., Type II error), or too large a sample size may waste resources and increase unnecessary participant risk. (4) In addition, quality assessment is a subjective process, and different authors may judge each factor differently, which may lead to differences in results compared with other reviews. Nevertheless, our review was assessed and checked by two independent authors.

This overview assessed various aspects of the included SRs/MAs using AMSTAR-2, PRISMA, GRADE, and ROBIS assessments, identifying areas for collective improvement. Firstly, it is crucial to provide a detailed and comprehensive description of the search strategy used in at least one literature database during the research process. Additionally, a list of excluded literature and the reasons for their exclusion should be provided to avoid biasing the research results and facilitate replication of the study. Secondly, it is important to assess reporting bias and provide evidence to support the evaluation results. Failing to do so may impact readers’ understanding and acceptance of the evaluation findings. Thirdly, it is recommended to develop and register a research plan in advance to enhance the rigor of the systematic review. Any potential conflicts of interest should also be clearly stated. The presence of competing interests can affect the authenticity and objectivity of the research results, thereby increasing the likelihood of publication bias. Fourth, it is advisable to broaden the language search scope and conduct comprehensive searches across major authoritative databases, research registries, and relevant gray literature, among other sources. Finally, further attention needs to be paid to the potential applications of acupuncture combined with other treatment modalities.

## 5 Conclusion

Based on the evidence collected, acupuncture shows promise as a treatment for PTN patients. However, it is important to note that the included SRs/MAs generally have low methodological quality and evidence quality. Therefore, caution must be exercised when interpreting this conclusion. To enhance future research in this area, it is recommended to adequately report methodological details and adhere to guidelines for conducting SRs/MAs.

## Data availability statement

The original contributions presented in the study are included in the article/Supplementary material, further inquiries can be directed to the corresponding author.

## Author contributions

H-xH: Methodology, Software, Writing – original draft, Investigation. Y-xL: Investigation, Methodology, Writing – original draft, Writing – review & editing. Y-sX: Methodology, Writing – original draft, Writing – review & editing. W-hF: Writing – review & editing, Funding acquisition. HX: Funding acquisition, Writing – review & editing, Methodology, Software, Supervision, Validation, Visualization, Writing – original draft.
