# Supplementary material for: The efficacy of acupuncture for trigeminal neuralgia: an overview of systematic reviews
Source: Front Neurol. 2024 Jul 5;15:1375587. doi: 10.3389/fneur.2024.1375587 (PMC11258042; doi:10.3389/fneur.2024.1375587)
Supplement: Supplementary file 1 [file Table_1.DOCX]

***Supplementary Table 1:*** The search strategy for each database

***Pubmed:***

#1 trigeminal neuralgia[Mesh] OR primary trigeminal neuralgia[Title/Abstract] OR idiopathic trigeminal neuralgia[Title/Abstract] OR classical trigeminal neuralgia[Title/Abstract]

#2 Acupuncture[Mesh] OR Acupuncture Therapy[Mesh] OR Meridians[Mesh]

#3 Acupuncture[Title/Abstract] OR Acupuncture Treatment*[Title/Abstract] OR Acupuncture Therapy[Title/Abstract] OR Acupuncture Therapies[Title/Abstract] OR Pharmacoacupuncture[Title/Abstract] OR Acupotomy[Title/Abstract] OR Acupotomies[Title/Abstract] OR Pharmacopuncture[Title/Abstract] OR Dry-needling[Title/Abstract] OR Body-acupuncture[Title/Abstract] OR Electroacupuncture[Title/Abstract] OR Electro-acupuncture[Title/Abstract] OR Auricular Acupuncture[Title/Abstract] OR Warm Needle[Title/Abstract] OR Meridian* [Title/Abstract] OR Ching Lo[Title/Abstract] OR Jing Luo[Title/Abstract] OR Jing mai[Title/Abstract] OR Jingluo[Title/Abstract] OR Jingmai[Title/Abstract]

#4 #2 OR #3

#5 Systematic Review [Publication Type] OR Meta-Analysis[Publication Type]

#6 Meta-Analysis as Topic[Mesh] OR Systematic Reviews as Topic[Mesh]

#7 Systematic Review as Topic[Title/Abstract] OR Systematic reviews as topic[Title/Abstract] OR Systematic Review*[Title/Abstract] OR Cochrane Review*[Title/Abstract] OR Systematic Evaluation*[Title/Abstract] OR Systematic Assessment*[Title/Abstract] OR Meta-Analysis as Topic[Title/Abstract] OR Meta Analysis as Topic[Title/Abstract] OR Meta-analytic*[Title/Abstract] OR Meta-analyses[Title/Abstract] OR Metaanalysis[Title/Abstract] OR Meta Analysis[Title/Abstract] OR Data Pooling*[Title/Abstract] OR Clinical Trial Overview*[Title/Abstract]

#8 #5 OR #6 OR #7

#9 #1 AND #4 AND #8

***The Cochrane Library***

#1 Mesh descriptor: [trigeminal neuralgia]explode all trees

#2 primary trigeminal neuralgia:ti,ab,kw OR idiopathic trigeminal neuralgia:ti,ab,kw OR

classical trigeminal neuralgia:ti,ab,kw

#3 #1 OR #2

#4 Mesh descriptor: [Acupuncture] explode all trees;

#5 Mesh descriptor: [Acupuncture Therapy] explode all trees;

#6 Mesh descriptor: [Meridians] explode all trees;

#7 "Acupuncture":ti,ab,kw or "Acupuncture Treatment*":ti,ab,kw or "Acupuncture Therapy":ti,ab,kw or "Acupuncture Therapies":ti,ab,kw or "Pharmacoacupuncture":ti,ab,kw or "Acupotomy":ti,ab,kw or "Acupotomies":ti,ab,kw or "Pharmacopuncture":ti,ab,kw or "Dry-needling":ti,ab,kw or "Body-acupuncture":ti,ab,kw or "Electroacupuncture":ti,ab,kw or "Electro-acupuncture":ti,ab,kw or "Auricular Acupuncture":ti,ab,kw or "Warm Needle":ti,ab,kw or "Meridian*":ti,ab,kw or "Ching Lo":ti,ab,kw or "Jing Luo":ti,ab,kw or "Jing mai":ti,ab,kw or "Jingluo":ti,ab,kw or "Jingmai":ti,ab,kw (Word variations have been searched)

#8 #4 OR #5 OR #6 OR #7

#9 Mesh descriptor: [Meta-Analysis as Topic] explode all trees;

#10 Mesh descriptor: [Systematic Reviews as Topic] explode all trees;

#11 "Systematic Review as Topic":ti,ab,kw or "Systematic reviews as topic":ti,ab,kw or "Systematic Review*":ti,ab,kw" or "Systematic Evaluation*":ti,ab,kw" or "Systematic Assessment*":ti,ab,kw" or "Cochrane Review*":ti,ab,kw" or "Meta-Analysis as Topic":ti,ab,kw or "Meta Analysis as Topic":ti,ab,kw or "Meta-analytic*":ti,ab,kw or "Meta-analyses":ti,ab,kw or "metaanalysis":ti,ab,kw or "Meta Analysis":ti,ab,kw or "Data Pooling*":ti,ab,kw or "Clinical Trial Overview*":ti,ab,kw (Word variations have been searched)

#12 #9 OR #10 OR #11

#13 #3 AND #8 AND #12

***Embase***

#1 'trigeminal neuralgia'/exp

#2 'primary trigeminal neuralgia':ti,ab,kw OR 'idiopathic trigeminal neuralgia':ti,ab,kw

OR 'classical trigeminal neuralgia':ti,ab,kw

#3 #1 OR #2

#4 'Acupuncture'/exp

#5 'Acupuncture':ab,ti OR 'Acupuncture Treatment*':ab,ti OR 'Acupuncture Therapy':ab,ti OR 'Acupuncture Therapies':ab,ti OR 'Pharmacoacupuncture':ab,ti OR 'Acupotomy':ab,ti OR 'Acupotomies':ab,ti OR 'Pharmacopuncture':ab,ti OR 'Dry-needling':ab,ti OR 'Body-acupuncture':ab,ti OR 'Electroacupuncture':ab,ti OR 'Electro-acupuncture':ab,ti OR 'Auricular Acupuncture':ab,ti OR 'Warm Needle':ab,ti OR 'Meridian*':ab,ti OR 'Ching Lo':ab,ti OR 'Jing Luo':ab,ti OR 'Jing mai':ab,ti OR 'Jingluo':ab,ti OR 'Jingmai':ab,ti

#6 #4 OR #5

#7 'Meta analysis'/exp OR 'Meta analysis (topic) '/exp OR 'Systematic review'/exp OR 'Systematic review (topic) '/exp

#8 'Systematic Review as Topic':ab,ti OR 'Systematic reviews as topic':ab,ti OR 'Systematic Review*':ab,ti OR 'Systematic Evaluation*':ab,ti OR 'Systematic Assessment*':ab,ti OR 'Cochrane Review*':ab,ti OR 'Meta-Analysis as Topic':ab,ti OR 'Meta Analysis as Topic':ab,ti OR 'Meta-analytic*':ab,ti OR 'Meta-analyses':ab,ti OR 'metaanalysis':ab,ti OR 'Meta Analysis':ab,ti OR 'Data Pooling*':ab,ti OR 'Clinical Trial Overview*':ab,ti

#9 #7 or #8

#10 #3 AND #6 AND #9

***Web of Science***

TS=(‘primary trigeminal neuralgia*’ OR ‘trigeminal neuralgia*’ OR ‘idiopathic trigeminal neuralgia*’ OR ‘classical trigeminal neuralgia*’) AND TS=(‘Acupuncture’ OR ‘Acupuncture Treatment*’ OR ‘Acupuncture Therapy’ OR ‘Acupuncture Therapies’ OR ‘Pharmacoacupuncture’ OR ‘Acupotomy’ OR ‘Acupotomies’ OR ‘Pharmacopuncture’ OR ‘Dry-needling’ OR ‘Body-acupuncture’ OR ‘Electroacupuncture’ OR ‘Electro-acupuncture’ OR ‘Auricular Acupuncture’ OR ‘Warm Needle’ OR ‘Meridian*’ OR ‘Ching Lo’ OR ‘Jing Luo’ OR ‘jing mai’ OR ‘Jingluo’ OR ‘jingmai’) AND TS=(‘Systematic Review as Topic’ OR ‘Systematic reviews as topic’ OR ‘Systematic Review*’ OR ‘Systematic Evaluation*’ OR ‘Systematic Assessment*’ OR ‘Cochrane Review*’ OR ‘Meta-Analysis as Topic’ OR ‘Meta Analysis as Topic’ OR ‘Meta-analytic*’ OR ‘Meta-analyses’ OR ‘Metaanalysis’ OR ‘Meta Analysis’ OR ‘Data Pooling*’ OR ‘Clinical Trial Overview*’)

***China National Knowledge Infrastructure***

SU=('三叉神经痛' +'三叉神经'+'原发性三叉神经痛') AND SU=('针刺'+'针灸'+'耳针'+'体针'+'舌针'+'电针'+'温针灸'+'腹针'+'头针'+'穴位'+'经络'+'经脉') AND SU=('荟萃分析'+'系统综述'+'系统评价'+'Meta分析')

***Wanfang Database***

(三叉神经痛 or 原发性三叉神经痛 or 三叉神经) and (针刺 or 针灸or耳针or体针or舌针or电针or温针灸or腹针or头针or 穴位or经络 or 经脉) and (荟萃分析 or 系统综述 or 系统评价 or Meta分析)

***Chongqing VIP***

(U=三叉神经痛 OR U=原发性三叉神经痛 OR U=三叉神经) AND (U=针刺 OR U=针灸 OR U=耳针 OR U=体针 OR U=舌针 OR U=电针 OR U=温针灸 OR U=腹针 OR U=头针 OR U=穴位 OR U=经络 OR U=经脉) AND (U=荟萃分析 OR U=系统综述 OR U=系统评价 OR U=Meta分析)

***Sino-Med***

1 "三叉神经痛"[不加权:扩展]

2 "针刺疗法"[不加权:扩展]

3 "针刺"[不加权:扩展]

4 "针灸疗法"[不加权:扩展]

5 "针刺"[常用字段:智能] OR "针灸"[常用字段:智能] OR "耳针"[常用字段:智能] OR "电针"[常用字段:智能] OR "体针"[常用字段:智能] OR "舌针"[常用字段:智能] OR "温针灸"[常用字段:智能] OR "头针"[常用字段:智能] OR "腹针"[常用字段:智能] OR "穴位"[常用字段:智能] OR "经脉"[常用字段:智能] OR "经络"[常用字段:智能]

6 (#2) OR (#3) OR (#4) OR (#5)

7 "Meta分析"[不加权:扩展]

8 "Meta分析"[常用字段:智能] OR "系统评价"[常用字段:智能] OR "荟萃分析"[常用字段:智能] OR "系统综述"[常用字段:智能]

9 (#7) OR (#8)

10 (#1) AND (#6) AND (#9)
